# Influence of DM-sensitivity on immunogenicity of MHC class II restricted antigens

**DOI:** 10.1136/jitc-2021-002401

**Published:** 2021-07-15

**Authors:** Anna Luise Bernhardt, Julia Zeun, Miriam Marecek, Hannah Reimann, Sascha Kretschmann, Judith Bausenwein, Edith D van der Meijden, Margarete M Karg, Tabea Haug, Lisa Meintker, Gloria Lutzny-Geier, Andreas Mackensen, Anita N Kremer

**Affiliations:** 1 Department of Internal Medicine 5 - Hematology and Internal Oncology, Friedrich-Alexander University Erlangen-Nuremberg, Erlangen, Bayern, Germany; 2 Schepens Eye Research Institute, Harvard Medical School, Boston, Massachusetts, USA

**Keywords:** antigens, CD4-positive T-lymphocytes, antigen presentation, immunotherapy, adoptive

## Abstract

**Background:**

Graft-versus-host-disease (GvHD) is a major problem in allogeneic stem cell transplantation. We previously described two types of endogenous human leukocyte antigen (HLA)-II restricted antigens depending on their behavior towards HLA-DM. While DM-resistant antigens are presented in the presence of HLA-DM, DM-sensitive antigens rely on the expression of HLA-DO-the natural inhibitor of HLA-DM. Since expression of HLA-DO is not upregulated by inflammatory cytokines, DM-sensitive antigens cannot be presented on non-hematopoietic tissues even under inflammatory conditions. Therefore, usage of CD4+ T cells directed against DM-sensitive antigens might allow induction of graft-versus-leukemia effect without GvHD. As DM-sensitivity is likely linked to low affinity peptides, it remains elusive whether DM-sensitive antigens are inferior in their immunogenicity.

**Methods:**

We created an in vivo system using a DM-sensitive and a DM-resistant variant of the same antigen. First, we generated murine cell lines overexpressing either H2-M or H2-O (murine HLA-DM and HLA-DO) to assign the two model antigens ovalbumin (OVA) and DBY to their category. Further, we introduced mutations within the two T-cell epitopes and tested the effect on DM-sensitivity or DM-resistance. Furthermore, we vaccinated C57BL/6 mice with either variant of the epitope and measured expansion and reactivity of OVA-specific and DBY-specific CD4+ T cells.

**Results:**

By testing T-cell recognition of OVA and DBY on a murine B-cell line overexpressing H2-M and H2-O, respectively, we showed that OVA leads to a stronger T-cell activation in the presence of H2-O demonstrating its DM-sensitivity. In contrast, the DBY epitope does not rely on H2-O for T-cell activation indicating DM-resistance. By introducing mutations within the T-cell epitopes we could generate one further DM-sensitive variant of OVA and two DM-resistant counterparts. Likewise, we designed DM-resistant and DM-sensitive variants of DBY. On vaccination of C57BL/6 mice with either epitope variant we measured comparable expansion and reactivity of OVA-specific and DBY-specific T-cells both in vivo and ex vivo. By generating T-cell lines and clones of healthy human donors we showed that DM-sensitive antigens are targeted by the natural T-cell repertoire.

**Conclusion:**

We successfully generated DM-sensitive and DM-resistant variants for two model antigens. Thereby, we demonstrated that DM-sensitive antigens are not inferior to their DM-resistant counterpart and are therefore interesting tools for immunotherapy after allogeneic stem cell transplantation.

## Background

CD4+ T cells are traditionally regarded as T-helper cells, which play a central role in orchestrating immune responses by providing help for maturation of dendritic cells, antibody production of B-cells and maintenance and induction of CD8+ T cells. Beyond that, CD4+ T cells have meanwhile been accepted to also mediate direct cytotoxicity and effector functions. Especially in tumor immunotherapy, it has been shown that CD4+ T cells are crucial^1^ and sometimes even sufficient to eradicate tumors in mice. In humans, it has been shown that CD4+ T cells can mediate graft-versus-leukemia (GvL) reactivity after allogeneic stem cell transplantation (aSCT) without induction of graft-versus-host-disease (GvHD).^2–4^ However, it has also been demonstrated that concomitant viral infection leading to upregulation of human leukocyte antigen (HLA) class II on non-hematopoietic cells induce severe GvHD after CD4+ donor lymphocyte infusion (DLI).^5^

We previously described that CD4+ T-cell responses after aSCT are directed against two different sets of HLA class II restricted antigens, that is, DM-sensitive and DM-resistant antigens.^6^ While DM-resistant antigens are presented on all HLA class II expressing cells, presentation of DM-sensitive antigens is abolished by expression of the non-classical HLA class II molecule HLA-DM and relies on co-expression of HLA-DO—the natural inhibitor of HLA-DM.^7^ In contrast to HLA-DM, which is co-regulated with the classical HLA class II molecules, HLA-DO is only expressed in B-cells, mature dendritic cells and thymic epithelial cells and is not upregulated by inflammatory cytokines.^6 8^ We have already shown that cytokine treated fibroblasts are not recognized by T-cells targeting DM-sensitive antigens,^6^ while most leukemic cells express sufficient HLA-DO to allow T-cell recognition.^9^ Therefore targeting DM-sensitive antigens might be promising in aSCT.

We identified three minor histocompatibility antigens with DM-sensitive properties directly ex vivo from a patient after aSCT and DLI for relapsed chronic myeloid leukemia,^10^ indicating their immunogenicity in vivo. However, it is widely accepted that HLA-DM favors the formation of high affinity HLA-peptide complexes. Furthermore, kinetic stability of HLA class II-peptide complexes has been shown to be a key parameter for immunodominance.^11^ In addition, immunodominance has also been directly linked to a DM-resistant phenotype.^12^ These, however, were mainly correlation studies using different sets of antigenic peptides. Therefore, the question rises whether DM-sensitive antigens can induce potent immune responses at all.

We here sought to explore, whether we could generate variants of the same T-cell epitope with contrary behavior towards HLA-DM (H2-M in mice) and use those to measure their immunogenicity in vivo. We show that by mutating single amino acid residues within the T-cell epitopes of murine DBY and the OT-2 epitope of OVA, we can actually induce a switch from DM-resistance to DM-sensitivity and vice versa. Furthermore, we demonstrate that the immunogenicity of these two epitope variants in vivo is comparable, leading to similar expansion and activation of T cells. Finally, we provide evidence that also in human natural immune responses against viral infections both, DM-sensitive and DM-resistant antigens, induce T-cell responses. In conclusion, our data indicate that DM-sensitive antigens are not inferior to DM-resistant antigens in regard to in vivo immune responses and are therefore promising targets for selective GvL effect after aSCT.

## Material and methods

### Hematopoietic cell isolation

Peripheral blood mononuclear cells (PBMC) were obtained from healthy individuals after approval by the Ethics committee of the Friedrich Alexander University Erlangen-Nuremberg and informed consent according to the Declaration of Helsinki. Mononuclear cells were isolated by Ficoll-isopaque separation and cryopreserved. CD4+ T cells and subsets were isolated by flow cytometric cell sorting. Epstein-Barr virus transformed lymphoblastic cell lines (EBV-LCL) were generated by magnetic bead isolation of CD19+ B cells according to the manufacturer’s instructions (Miltenyi Biotec) and addition of EBV viral supernatant (B95.8).

### Cell culture

291PC,^13^ A20 (ATCC, TIB-208), Phoenix-A (ATCC, CRL-3213), and EBV-LCL were cultured in RPMI 1640 (PAN-Biotech) with 10% FCS, 40 IU/mL penicillin, 40 µg/mL streptomycin, 2 mM l-glutamine, 0.4% vitamin solution, 50 µM β-mercaptoethanol, 1% minimal essential media, and 1 mM sodium pyruvate (all Gibco, Thermo Fisher Scientific). In murine splenocyte culture 50 IU/mL recombinant interleukin (IL)-2 (Proleukin) were added. For in vitro expansion of Marilyn-derived CD4+ splenocytes, cells were activated in anti-CD3 (3 µg/mL; 145–2 C11; Biolegend) and anti-CD28 (2 µg/mL; 37.51; Biolegend) coated plates in the presence of 5 IU/mL IL-2 for 24 hours, harvested and subsequently expanded with addition of 10 IU/mL IL-15 (Biolegend) for 7 days.^14^ To generate immature dendritic cells monocytes were isolated by adherence and cultured for 5 days in the presence of IL-4 (500 IU/mL; Peprotech), GM-CSF (100 IU/mL; Miltenyi) and TGF-beta (5 ng/mL; Peprotech).

Human T-cells were cultured in RPMI 1640, as mentioned, but with 5% human serum (PAN-Biotech), 5% FCS, and 100 IU/mL IL-2 (Proleukin). Every 11–14 days, T cells were restimulated with 50 Gy irradiated allogeneic feeder cells and 0.8 µg/mL phytohemagglutinin (PHA) (Oxoid), as previously described.^15^ Culture supernatants of cell lines were regularly tested for mycoplasma contamination (Minerva Biolabs) by PCR.

### Flow cytometry and cell sorting

Flow cytometric analyses were performed on a FACSCanto II (BD Biosciences), and cell sorting was carried out on a FACSAria II SORB (BD Biosciences) using FITC-labeled human α-CD56 (NCAM16.2; BD), PE-labeled α-IFN-γ (AN.18.17.24; Miltenyi Biotec), α-NGFR (C40-1457; BD), α-CD45RO (UCHL1; BD) and α-CD69 (FN50; BD), APC-labeled α-TCRα2 (B20.1; BD), α-TCRβ6 (RR4-7; Biolegend), α-CD137 (4B4-1; BD), APC-labeled and BV421-labeled α-human CD4 (RPA-T4; BD and Biolegend), BV421-labeled α-CCR7 (2-L1-A; BD), PerCP-labeled α-murine CD4 (RM4-5; BD), PE-Cy7 labeled α-TNF (MP6-XT22; BD) and α-Ki67 (16A8; Biolegend), VioGreen labeled α-murine CD8 (53–6.7; Miltenyi Biotec), VioBlue labeled α-CD45.1 (A20; Miltenyi Biotec) and eFluor450 labeled α-TCRβb5 (MR9-4; eBioscience) and α-I-A^b^ (AF6-120.1; eBioscience).

For intracellular staining of human HLA-DM/HLA-DO and murine H2-M/H2-O the following antibodies were used: Alexa 647 labeled anti-human HLA-DO (Mags.DO5), anti-mouse H2-Oβ (Mags.Ob1) (both kindly provided by Lisa Denzin; Rutgers, The State University of New Jersey), PE-labeled anti-human HLA-DM (Ma.P.DM1; BioLegend), anti-rat IgG1 secondary antibody (MRG1-58, BioLegend), mouse IgG1 isotype control (MOPC-21, BioLegend), and purified anti-mouse H2-M (2E5A, BD). 1×10^6^ cells were incubated with Zombie NIR (BioLegend) for 30 min at 4°C in the dark to stain dead cells. After washing with PBS cells were fixed with 1% paraformaldehyde for 8 min at 4°C in the dark. For permeablisation fixed cells were incubated with saponine buffer (PBS with 2 %BSA and 0.1% saponine) for 15 min at 4°C in the dark and finally stained for 30 min at 4°C in the dark.

For intracellular cytokine staining 10 µg/mL Brefeldin A (Sigma-Aldrich) was added and cell co-cultures were incubated for 14–20 hours at 37°C. Subsequently, surface markers were stained for 20 min at 4°C, cells were fixated with 1% formaldehyde for 8 min at 4°C, permeabilized using 0.1% saponine and 2% BSA in PBS for 15 min at 4°C and intracellular targets stained for 30 min at 4°C.

To quantify specific T-cells from blood, 10 µl of murine blood was mixed with indicated antibodies in FcγRIII/RII blocking solution and incubated for 30 min at 4°C. Subsequently, erythrocytes were lysed by Ammonium-Chloride-Potassium (ACK) buffer (155 mM CIH_4_N; 0.1 mM EDTA; 10 mM KHCO_3_) for 5 min and cell count beads (Beckman Coulter) were added before analysis in flow cytometry.

### Retroviral constructs and transduction

Human HLA-DOα/β and HLA-DMα/β, murine H2-Oα/β and H2-Mα/β and murine I-A^b^ with α-chain and β-chain were all cloned into the pMP71^16^ retroviral vector. For α-chains truncated nerve growth factor receptor (ΔNGFR) was used as marker gene, for β-chains green fluorescent protein (GFP). I-A^b^ α-chains and β-chains were cloned into the same vector, linked by an internal ribosome entry site (IRES), and surface expression of I-A^b^ was used as marker. All oligonucleotides are provided in the Supplemental Materials (online supplemental table 1). Phoenix-A cells were transfected as previously described^17^ with X-tremeGENE HP DNA Transfection Reagent (Roche). EBV-LCL, 291PC, and A20 cell lines were transduced on culture plates coated with 30 µg/mL recombinant human fibronectin (Takara Shuzo). Transduction efficiency was measured by flow cytometry with α-human CD271/ΔNGFR and α-I-A^b^ antibodies or GFP expression and transduced cells were isolated based on marker gene expression by flow cytometric cell sorting. All experiments involving retroviral vectors were approved by the government and handled according to biosafety level 2.

10.1136/jitc-2021-002401.supp1 Supplementary data

### Quantitative real-time PCR

Total RNA was isolated from cell lines and complementary DNA synthesized with M-MuLV reverse transcriptase (New England Biolabs GmbH). Quantitative real-time PCR was performed on a StepOnePlus Real-Time PCR system (Applied Biosystems). PCR was performed in 20 µl reactions with 100 nM of each primer (online supplemental table 2) and SYBR select master mix (Applied Biosystems). Expression of target genes was normalized to the housekeeping gene 18 S ribosomal RNA and calculated using the comparative threshold cycle (ΔΔCT) method.^18^

### Mice

T-cell receptor transgenic Marilyn mice (C57BL/6) were kindly provided by Olivier Lantz (Institute Curie, Paris, France).^19^ Wildtype C57BL/6 and anti-ovalbumin (OVA) T-cell receptor transgenic OT-2 mice (C57BL/6) were kindly provided by Armin Gerbitz (Princess Margaret Cancer Center, Toronto, Canada). All mice were kept under specific pathogen-free conditions following institutional guidelines.

### Vaccination and adoptive transfer

In vaccination studies, CD4+ T cells from splenocytes of OT-2 or Marilyn mice were isolated by magnetic cell separation using the CD4 untouched kit (Miltenyi Biotec). Wildtype C57BL/6 mice were intraperitoneally (i.p.) injected with 4×10^6^ OT-2 specific or 2×10^6^ DBY-specific T-cells after in vitro expansion. Twenty-four hours later, mice were subcutaneously vaccinated with 10 µg of the indicated 30mer peptides using 50 µg Poly(I:C) or CpG with 50 µl IFA as adjuvant. Epitope-specific T cells were quantified from blood of vaccinated mice based on expression of CD4+/TCRvα2/β5+ (OT-2) or CD4+/TCRvβ6+/CD45.1+ (Marilyn). Cell numbers were quantified using flow count fluorospheres for normalization (Beckman Coulter). On day 7 after vaccination mice were euthanized and cells from spleen and lymph nodes were isolated for analysis.

### Antigen-presentation assays

To analyze antigen presentation, irradiated (50 Gy) stimulator cells (1×10^4^ cells/well) were cocultured with CFSE-stained OT-2 or Marilyn T-cells (2×10^4^ cells/well) for 3–5 days. CFSE staining was performed at 5 µM for 5 min at room temperature. T-cell activation was assessed either by proliferation as measured by dilution of CFSE or by cytokine production of tumor necrosis factor (TNF) as measured by intracellular flow cytometric staining or interferon (IFN)-γ in ELISA according to the manufactures’ instructions (BD OptEIA). To measure antigen presentation of the different peptide variants, indicated peptides were loaded with 5 µg/mL (OVA) or 0.5 µg/mL (DBY) (if not otherwise stated) on 291PC or A20 cell lines or primary CFSE-stained splenocytes. All peptides were synthesized at Genecust and are listed in online supplemental tables 1 and 2.

For retesting of splenocytes and lymph node-derived T-cells from vaccinated mice, spleen and lymph nodes were isolated on day 7 after vaccination. Cells were stained with CFSE and stimulated with indicated concentrations of 30mer OVA or DBY peptides. T-cell activation was measured by CFSE dilution, ELISA and intracellular cytokine staining as indicated in the respective figures.

### Human T-cell libraries

Total CD4+ T cells or T-cell subsets (CCR7+/CD45RO– for naïve T-cells, CCR7–/CD45RO+ for effector memory cells, CCR7+/CD45RO+ for central memory cell and CCR7–/CD45RO– for effector memory RA cells) were isolated by flow cytometric cell sorting from PBMC of healthy donors and plated with 250 cells/well in 96 well plates. T cells were non-specifically expanded using irradiated (50 Gy) allogeneic PBMC, PHA (0.8 µg/mL) and IL-2 (100 IU/mL). After 2–3 weeks of expansion, T-cell pools were tested for recognition of autologous EBV-LCL overexpressing HLA-DM or HLA-DO with or without prior loading of 1.0 µg/mL cytomegalovirus (CMV) antigen (Jena Bioscience). T-cell recognition was measured by IFN-γ ELISA (eBioscience).

### Isolation of EBV and CMV specific T-cell clones

T-cells of healthy donors were stimulated with autologous EBV-LCL with or without loaded CMV antigen. Activated CD4+ T cells were isolated by single cell sorting based on expression of CD4 and T-cell activation markers CD137 and CD69. Isolated T-cell clones were expanded using irradiated (50 Gy) allogeneic PBMC, PHA (0.8 µg/mL) and IL-2 (100 IU/mL). Growing T-cell clones were tested for specific recognition of autologous EBV-LCL overexpressing HLA-DM or HLA-DO with or without prior CMV antigen loading.

In order to obtain CMV-specific T-cell clones PBMCs of healthy donors were stimulated with a pool of 17 CMV-derived peptides^20^ at a concentration of 1 µg/mL per peptide for 2 hours, thoroughly washed and cultured for 10 days. T cells were restimulated with autologous immature dendritic cells pulsed with the same pool of CMV peptides for 16 hours and activated T-cells were isolated by single cell sorting based on the expression of CD4+/CD69+/CD137+. Isolated T-cell clones were expanded using irradiated allogeneic PBMC, PHA and IL-2 as described above.

### Statistics

All graphs were created, and statistical analyses were performed using Prism 8 (GraphPad Software). Kruskal-Wallis test for non-parametric data was used for multiple comparisons. Two-tailed Mann-Whitney U test was used to compare two groups wherever indicated. For comparison of frequencies Pearson’s χ^2^ or for small numbers Fisher’s exact test were used. For statistical analyses, at least three individual experiments were performed and significance indicated. Illustrated experiments are representatives of at least three independent experiments unless otherwise stated.

## Results

### Model antigens OVA and DBY display differential behavior towards H2-M

**Figure 1:**
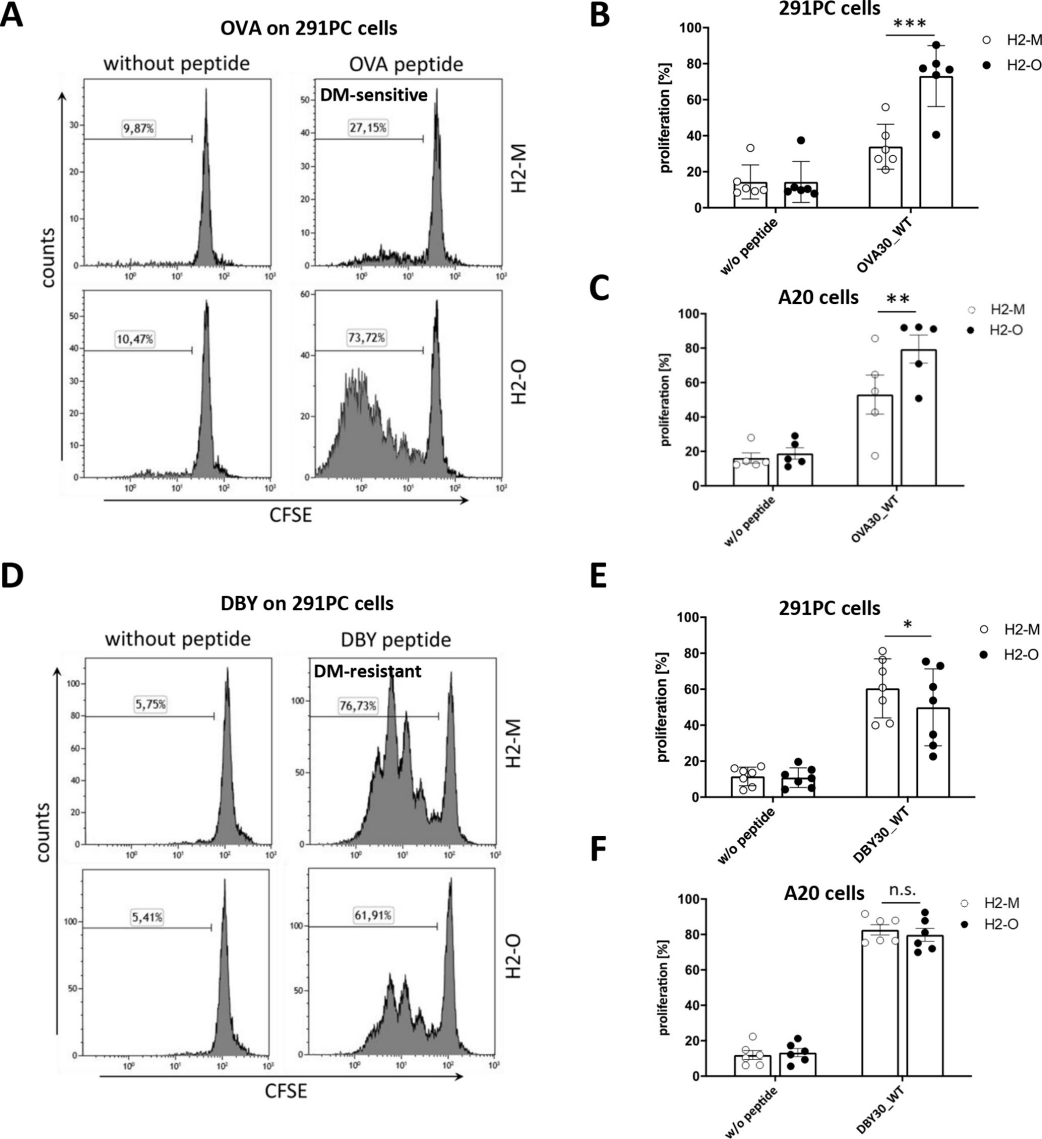
CD4+ T-cell epitopes of OVA and DBY display differential behavior towards H2-M. CD4+TCRvα2/β5+ T-cells derived from OT-2 mice were stained with CFSE and co-cultured with irradiated OVA peptide loaded 291PC (A, B) or A20 (C) cells transduced with H2-M or H2-O. T-cell proliferation on day 5 is depicted as measured by flow cytometry. CD4+ TCRvβ6+ T-cells derived from Marilyn mice were stained with CFSE and co-cultured with DBY peptide loaded 291PC (D, E) or A20 (F) cell lines overexpressing H2-M or H2-O. T-cell proliferation on day 3 was measured as indicator for peptide presentation. Data are shown as mean±SEM of n=5–6 individually performed experiments. Panel A and D show representatives of six to seven independent experiments. A data summary of the six to seven individually performed experiments is shown in B and E. Statistical differences were calculated using paired t-test. *p<0.05; **p<0.01; ***p<0.001. ns, not significant; OVA, ovalbumin; WT, wildtype.

In order to compare immunogenicity of DM-sensitive and DM-resistant epitopes without the confounder of different T-cell receptors, we sought to create two variants of the same CD4+ T-cell epitope with contrary behavior towards H2-M using two model antigens. For this purpose, we used the OT-2 epitope of OVA^21^ and the Marilyn epitope of murine DBY.^19^ First, we assessed their natural behavior towards H2-M. Therefore, we established a cell line model based on two murine lymphoma cell lines (291PC and A20), which were retrovirally transduced with the class II (I-A^b^) restriction molecule for OVA and DBY as well as H2-M or H2-O. Overexpression of H2-M and H2-O was confirmed by direct staining of the overexpressed protein in flow cytometry, qPCR as well as marker gene expression in flow cytometry (online supplemental figure 1). In addition, equivalent surface expression of the restriction molecule I-A^b^ was confirmed by flow cytometry in all cell lines (online supplemental figure 1C, D). We then pulsed H2-M and H2-O expressing 291PC and A20 cell lines with 30mer peptides containing either the OT-2 epitope of OVA (ISQAVHAAHAEINEAGREVVGSAEAGVDAA) or the Marilyn epitope of murine DBY (YRQSSGSANAGFNSN4RANSSRSSGSSHNRG) and tested T-cell recognition by co-incubating peptide loaded cell lines with the epitope-specific T-cells. As depicted in figure 1A, B, the OVA pulsed H2-O transduced 291PC cell line lead to strong proliferation of OT-2 T-cells, while the same peptide did hardly induce any T-cell proliferation when pulsed on H2-M transduced cells. This shows that the OT-2 epitope of OVA has a DM-sensitive phenotype thereby confirming earlier studies demonstrating that the OT-2 epitope of OVA is better presented in wildtype as compared with H2-O -/- B cells.^22 23^ This could also be reproduced using the A20 cell line (figure 1C). In contrast, the Marilyn epitope of murine DBY induced specific T-cell proliferation on loading on both H2-M and H2-O transduced 291PC cells, with slightly stronger responses on H2-M transduced cells, indicating a DM-resistant phenotype (figure 1D, E). These results were also reproducible using the A20 cell line (figure 1F).

10.1136/jitc-2021-002401.supp2 Supplementary data

In conclusion, these experiments show that the OT-2 epitope of OVA is DM-sensitive, whereas the Marilyn epitope of murine DBY is DM-resistant.

### Single amino acid residues decide about DM-sensitivity

To switch the behavior towards H2-M we mutated the amino acid sequence of the OVA epitope as depicted in table 1. The amino acid substitutions were based on binding affinity prediction by the NetMHCII 2.2 server as well as described binding interactions of peptides with the I-A^b^ restriction molecule.^21 24 25^

**Table 1:** Ovalbumin wildtype and mutated variant peptides

Peptide	Sequence	NetMHCII 2.2 prediction	
		Binding	Affinity (nM)
WT	ISQA**V^-2^HA^1^AHAEI^6^N**EAGREVVGSAEAGVDAA	weak	339
I334A	ISQA**V^-2^HA^1^AHAEA^6^N**EAGREVVGSAEAGVDAA	weak	89
A329Y/I334A	ISQA**V^-2^HY^1^AHAEA^6^N**EAGREVVGSAEAGVDAA	strong	37
V327F/A329Y/I334A	ISQA**F^-2^HY^1^AHAEA^6^N**EAGREVVGSAEAGVDAA	strong	14

Anchor amino acids of the core peptide region which possibly interact with the I-A^b^ restriction molecule are indicated by numbers. Mutated amino acids are highlighted. Binding strength is characterized by the binding affinity as calculated by the NetMHCII 2.2 software (strong <50 nM; weak <500 nM).

**Figure 2:**
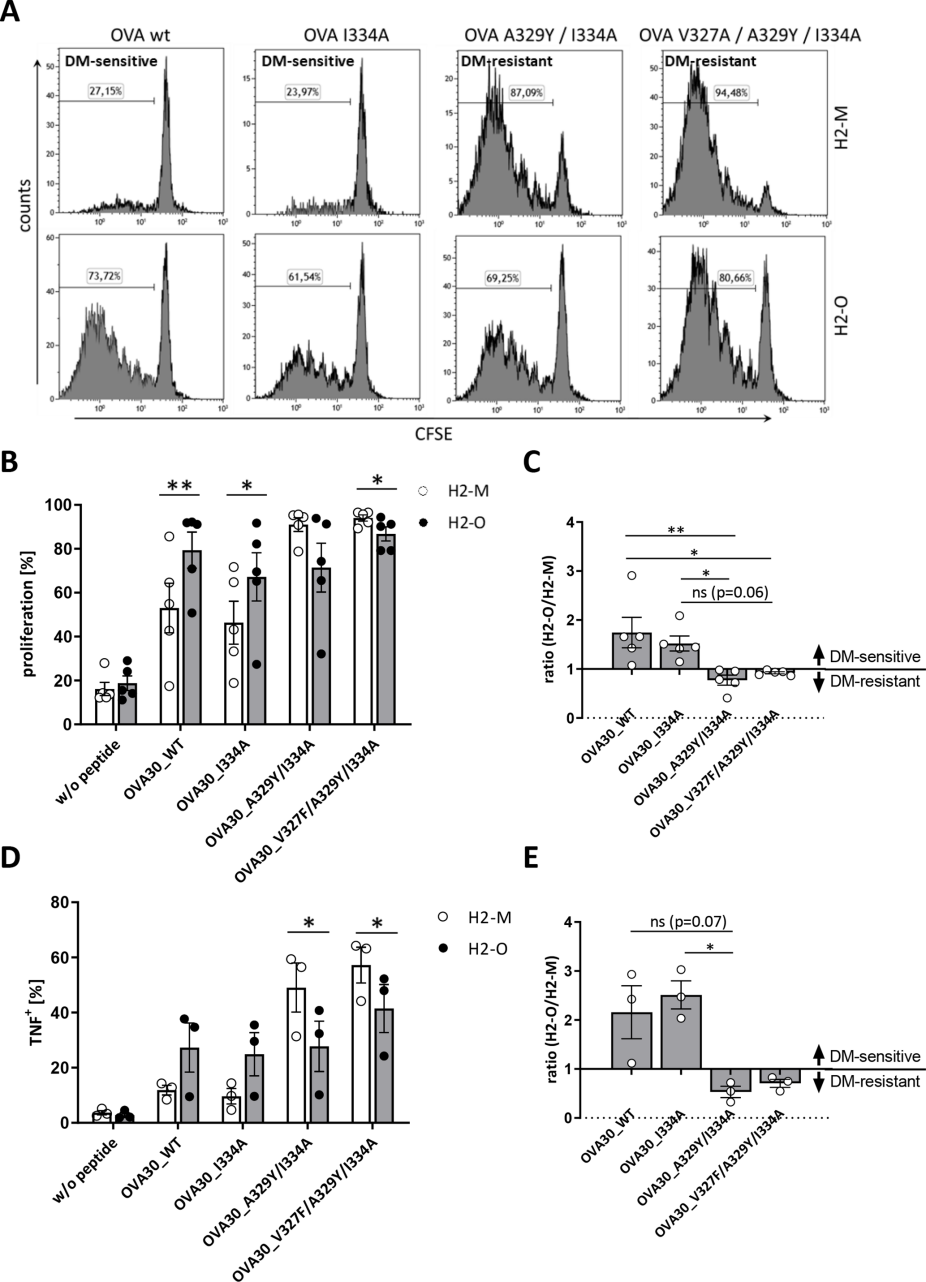
OVA-derived OT-2 epitope can be switched from DM-sensitive to resistant. CD4+TCRvα2/β5+ T-cells derived from OT-2 mice were stained with CFSE and co-cultured with irradiated 291PC (A) or A20 (B) cells transduced with H2-M or H2-O and loaded with the indicated peptides. For (A) one representative of six independent experiments is shown. Proliferation was measured on day 5 using flow cytometry. (C) Depicted are proliferation data from panel B as ratio of H2-O/H2-M expressing cells as stimulators. Ratios >1 are defined as DM-sensitive, ratios <1 as DM-resistant. Illustrated are means±SEM (n=5). (D) Intracellular TNF production was measured after co-culture with A20 cells on day 3. (E) Shown are intracellular TNF data from panel D as ratio of H2-O/H2-M expressing cells as stimulators. Ratios >1 are defined as DM-sensitive, ratios <1 as DM-resistant. Illustrated are means±SEM (n=3). Statistical differences were calculated using paired t-test (B, D). Significance for the ratios in panel C and E was calculated using Kruskal-Wallis test for non-parametric data with multiple comparisons. *p<0.05; **p<0.01. ns, not significant; OVA, ovalbumin; TNF, tumor necrosis factor; WT, wildtype.

Proliferation of OT-2 T-cells after incubation with peptide loaded H2-M or H2-O transduced 291PC cell lines showed that the OVA I334A mutation displayed DM-sensitive characteristics just as the wildtype epitope. In contrast, the double-mutant OVA A329Y/I334A as well as the triple mutant OVA V327A/A329Y/I334A were clearly switched to a DM-resistant phenotype (figure 2A and online supplemental figure 2A). These results could be reproduced using the A20 cell line measuring both, T-cell proliferation and intracellular TNF (figure 2B–E and online supplemental figure 2B).

Likewise, we sought to switch the DBY epitope from DM-resistant to DM-sensitive. We generated 12 different mutated variants of this epitope, as depicted in table 2.

**Table 2:** DBY wildtype and mutated variant peptides

Peptide	Sequence	NetMHCII 2.2. prediction	
		Binding	Affinity (nM)
WT	YRQSSGSAN**A^-2^GF^1^NSN^4^RA^6^NSS^9^**RSSGSSHNRG	Weak	158
A10F/N15A	YRQSSGSAN**F^-2^GF^1^NSA^4^RA^6^NSS^9^**RSSGSSHNRG	Strong	17
F12W	YRQSSGSAN**A^-2^GW^1^NSN^4^RA^6^NSS^9^**RSSGSSHNRG	–	569
A10F/F12A	YRQSSGSAN**F^-2^GA^1^NSN^4^RA^6^NSS^9^**RSSGSSHNRG	–	1030
F12I	YRQSSGSAN**A^-2^GI^1^NSN^4^RA^6^NSS^9^**RSSGSSHNRG	–	1361
F12M	YRQSSGSAN**A^-2^GM^1^NSN^4^RA^6^NSS^9^**RSSGSSHNRG	–	2182
F12A	YRQSSGSAN**A^-2^GA^1^NSN^4^RA^6^NSS^9^**RSSGSSHNRG	–	2321
F12A/A17P	YRQSSGSAN**A^-2^GA^1^NSN^4^RP^6^NSS^9^**RSSGSSHNRG	–	2460
F12A/R20M	YRQSSGSAN**A^-2^GA^1^NSN^4^RA^6^NSM^9^**RSSGSSHNRG	–	2608
F12A/R20T	YRQSSGSAN**A^-2^GA^1^NSN^4^RA^6^NST^9^**RSSGSSHNRG	–	2852
A10F/F12A/N18L	YRQSSGSAN**F^-2^GA^1^NSN^4^RA^6^LSS^9^**RSSGSSHNRG	–	3301
F12G	YRQSSGSAN**A^-2^GG^1^NSN^4^RA^6^NSS^9^**RSSGSSHNRG	–	5440
F12A/A17I	YRQSSGSAN**A^-2^GA^1^NSN^4^RI^6^NSS^9^**RSSGSSHNRG	–	5862

Anchor amino acids of the core peptide region which possibly interact with the I-A^b^ restriction molecule are indicated by numbers. Mutated amino acids are highlighted. Binding strength is characterized by the binding affinity as calculated by the NetMHCII 2.2 software (strong <50 nM; weak <500 nM). Peptide variants that did not induce any T-cell recognition are shaded in gray.

**Figure 3:**
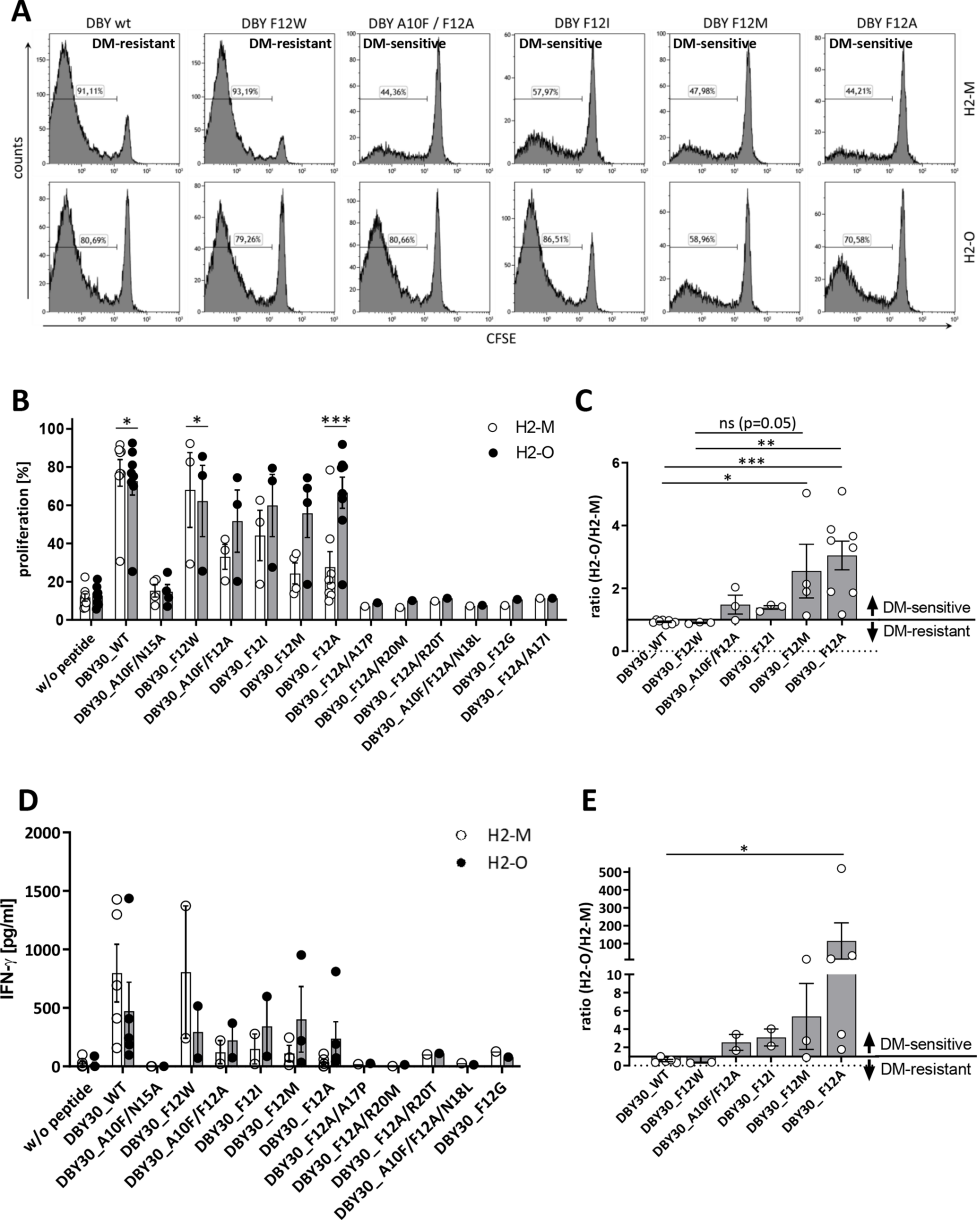
Mutation of DBY induces DM sensitivity. CD4+TCRvβ6+ T-cells derived from Marilyn mice were stained with CFSE and cocultured with irradiated 291PC (A) or A20 (B) cells transduced with H2-M or H2-O and loaded with the indicated peptides. Proliferation was measured on day 3 using flow cytometry. For (A) one representative of six independent experiments is shown. (C) Depicted are proliferation data from panel B as ratio of H2-O/H2-M expressing cells as stimulators. Ratios >1 are defined as DM-sensitive, ratios <1 as DM-resistant. Means±SEM are indicated (n≥3). (D) IFN-γ secretion was measured by ELISA after co-culture with A20 cells by day 2. (E) Shown are IFN-γ data from panel D as ratio of H2-O/H2-M expressing cells as stimulators. Ratios >1 are defined as DM-sensitive, ratios <1 as DM-resistant. Demonstrated are means±SEM (n≥2). Statistical differences were calculated using paired t-test (B, D). Significance for the ratios in panel C and E was calculated using Kruskal-Wallis test for non-parametric data with multiple comparisons. *p<0.05; **p<0.01; ***p<0.001. IFN, interferon; ns, not significant; WT, wildtype.

Seven of these peptides were not able to activate the DBY-specific T-cells (A10F/N15A, F12A/A17P, F12A/R20M, F12A/R20T, A10F/F12A/N18L, F12G, and F12A/A17I). In addition to the wildtype peptide, we identified also one mutated variant (F12W) with a DM-resistant phenotype. The four remaining mutants (A10F/F12A, F12I, F12M, and F12A) were switched to DM-sensitive (figure 3A and online supplemental figure 2C). These results could be corroborated in the A20 cell line by measuring proliferation and IFN-γ secretion by ELISA (figure 3B–E and online supplemental figure 2D).

In summary, by mutating one to three amino acid residues within the epitopes of OVA and DBY, we were able to generate both DM-sensitive and DM-resistant antigen variants of both epitopes.

### Immunogenicity of DM-sensitive and DM-resistant variants is comparable in vivo

**Figure 4:**
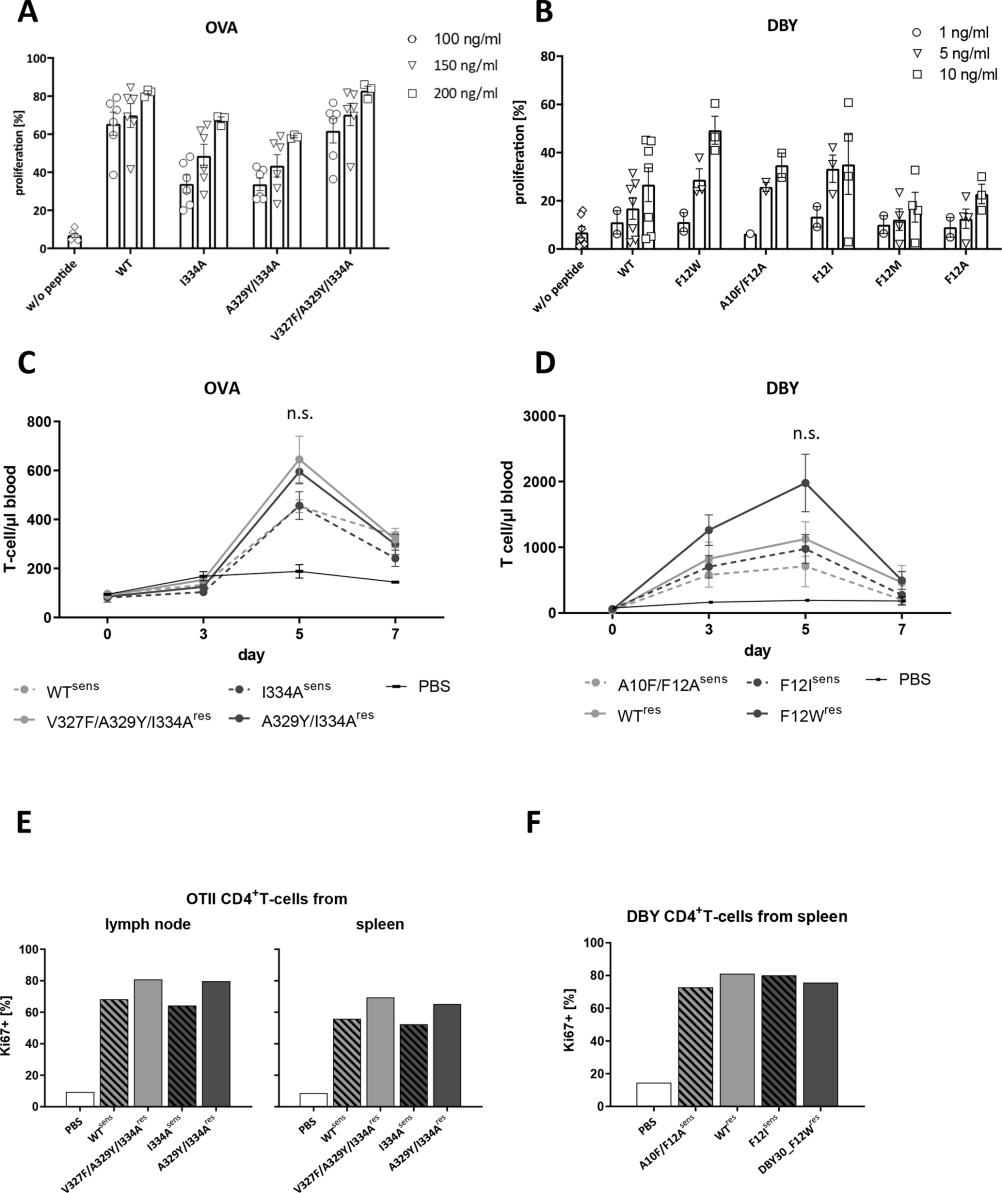
DM-sensitive and DM-resistant peptides induce comparable T-cell proliferation in vivo (A) CFSE-stained OT-2-derived splenocytes of at least three different donors were loaded with indicated amounts of peptides and proliferation of CD4+TCRvα2/β5+ T-cells was measured on day 3. (B) Marilyn-derived splenocytes of three different mice were stained with CFSE and loaded with indicated amounts of peptides. Proliferation of CD4+TCRvβ6+ T-cells was measured on day 2. (C) CD4+TCRvα2/β5+ OT-2 T-cells were injected i.p. in C57BL/6 mice and mice were vaccinated with the indicated peptides the next day. Absolute numbers of the CD4+TCRvα2/β5+ T-cells were measured in peripheral blood of treated mice. Two-tailed Mann-Whitney U test was used to compare proliferation data by day 5. Data show means±SEM (n=3–4 mice/group). ns: not significant. (D) C57BL/6 mice were injected i.p. with in vitro expanded CD4+TCRvβ6+ Marilyn-derived T-cells and subsequently vaccinated with the indicated peptides. Absolute numbers of the CD4+TCRvβ6+/CD45.1+ T-cells were measured in peripheral blood of treated mice (n=3–4 mice/group). Two-tailed Mann-Whitney U test was used to compare proliferation data by day 5. ns: not significant. (E) Splenocytes and cells from lymph nodes were isolated from mice 7 days after transfer of T-cells. Proliferative capacity of CD4+TCRvα2/β5+ T-cells was measured by expression of intracellular Ki-67. Illustrated are pooled data of groups of three to four mice. (F) Splenocytes were isolated from mice after vaccination and i.p. transfer of Marilyn T-cells. Proliferative capacity of CD4+TCRvβ6+ T-cells was measured by intracellular detection of Ki-67. Illustrated are pooled data of groups of three to four mice. i.p., intraperitoneally.

Before testing immunogenicity of our antigen variants in vivo, we titrated all variants on murine splenocytes to get an idea of their immunogenic capacity. For the OT-2 epitope, we found one DM-sensitive (wt) and one DM-resistant (V327F/A329Y/I334A) variant with a comparable capacity of T-cell activation. The other two variants (DM-sensitive: I334A and DM-resistant: A329Y/I334A) induced a little bit lower T-cell proliferation, but were comparable between each other (figure 4A). Based on these results, we compared in our in vivo studies these two pairings, that is, DM-sensitive (wt) versus DM-resistant (V327F/A329Y/I334A) and DM-sensitive (I334A) versus DM-resistant (A329Y/I334A).

Likewise, we compared the T-cell activation capacity of the generated DBY peptide variants (figure 4B). Two of the variants (F12M and F12A) elicited lower T-cell proliferation and were not included in further analysis. The other four were paired as follows: DM-sensitive (A10F/F12A) versus DM-resistant (wt) and DM-sensitive (F12I) vs DM-resistant (F12W).

To compare our epitope variants in vivo, we injected C57BL/6 mice with OT-2 or Marilyn T-cells i.p. followed by subcutaneous vaccination with the respective peptides 24 hours after cell transfer. Subsequently, we measured in vivo proliferation of the injected epitope-specific T-cells on days 3, 5 and 7 after transfer. Epitope-specific T-cells were quantified from peripheral blood based on expression of the transgenic TCR (vα2/β5+for OT-2 or vβ6+/CD45.1+for DBY). Cell numbers were quantified using flow count fluorospheres for normalization. For all four OVA peptides, we observed an increase in T-cell numbers on day 5 after T-cell transfer and a decline on day 7. Although there was a trend towards slightly higher T-cell numbers after vaccination with the two DM-resistant peptides in the Poly(I:C) group (figure 4C and online supplemental figure 3A), this did not reach significance (p=0.09) and was not reproducible with CpG as adjuvant (online supplemental figure 4A). There was also a trend towards higher proliferative capacity as measured by Ki67 expression after vaccination with DM-resistant peptides. This was consistent both with CpG and Poly(I:C) in T-cells isolated from spleen and lymph nodes (figure 4E and online supplemental figure 4B). Nevertheless, these data have to be interpreted with caution as the absolute number of total OT-2 T-cells were very limited within the splenocyte population and cells from individual mice within one group had to be pooled. Total CD8+ T cells were not affected by vaccination (online supplemental figure 3C).

Likewise, we vaccinated C57BL/6 mice with the different DBY peptides. Here we already observed proliferation of the specific CD4+ T-cells on day 3 after transfer and a further increase on day 5. On day 7, levels in peripheral blood were almost back to baseline. Similar as detected for the OVA antigen, we observed a trend towards higher numbers for the two DM-resistant peptides, but again, this was not significant (p=0.05) (figure 4D and online supplemental figure 3B). When we again measured proliferative capacity of the T-cells isolated from spleen by day 7, we did not observe any difference between the four peptides (figure 4F). Also here, CD8+ T cells were not affected by vaccination (online supplemental figure 3D).

We further isolated the specific T-cells from spleens of OVA or DBY vaccinated mice on day 7 after T-cell transfer and re-tested these cells ex vivo against the original vaccination peptide as well as the counterpart peptide. We measured proliferation and intracellular TNF on stimulation with titrated concentrations of the individual peptide. Neither for OVA nor for DBY we could observe any significant differences in functional avidity of the T-cells (online supplemental figure 5).

In summary, our in vivo vaccination studies with OVA and DBY revealed that DM-resistant and DM-sensitive peptides have comparable immunogenic abilities.

### DM-sensitive antigens can induce T-cell reactivities in natural immune responses in humans

**Figure 5:**
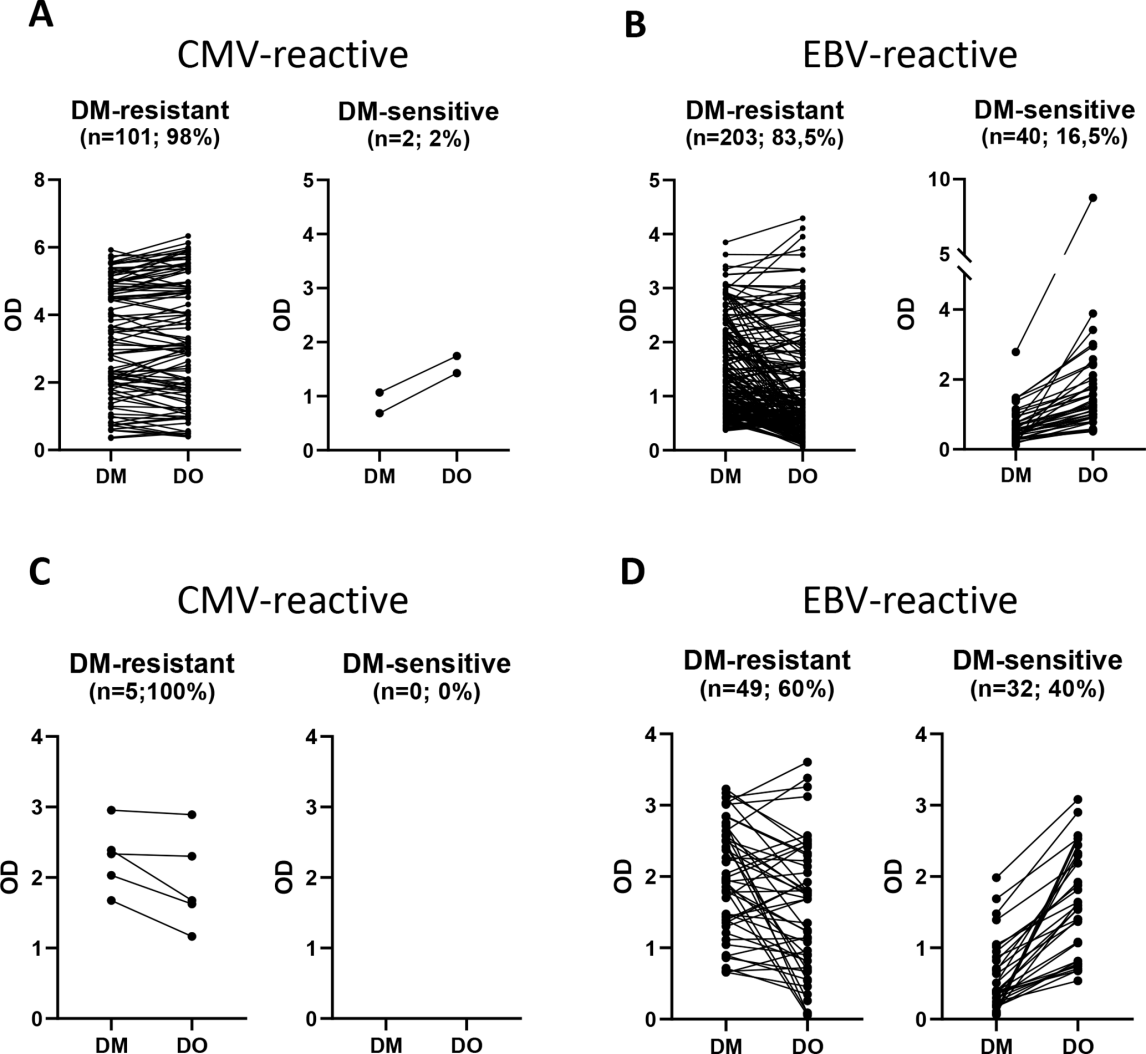
DM-sensitive and DM-resistant antigens can induce T-cell reactivity against viral antigens in humans (A, B) CD4+T cells of healthy donors were isolated and non-specifically expanded. T-cell lines were tested for reactivity against autologous EBV-LCL overexpressing HLA-DM or HLA-DO. Pools with no or low reactivity (OD_(450-570)_<0.5) were assigned as ‘non-reactive’. The remaining pools were categorized as DM-sensitive or DM-resistant based on the differential recognition of EBV-LCL with HLA-DM or HLA-DO. Pools with >50% increased reactivity on expression of HLA-DO were assigned as DM-sensitive. ‘Non-reactive’ pools were retested against autologous EBV-LCL overexpressing HLA-DM or HLA-DO loaded with CMV antigen. Again pools with no or low reactivity (OD_(450-570)_<0.5) were assigned as ‘non-reactive’. The remaining pools were categorized as DM-sensitive or DM-resistant based on the differential recognition of EBV-LCL with HLA-DM or HLA-DO loaded with CMV antigen. Pools with >50% increased reactivity on expression of HLA-DO were assigned as DM-sensitive. n: number of reactive T-cell lines. CD4+ T cell libraries were generated from eight individual healthy donors. (C, D) CD4+ T-cells of healthy donors were stimulated with autologous EBV-LCL with or without CMV antigen and activated T-cells were clonally isolated and expanded. For EBV, we generated 81 different clones, for CMV five clones. All clones were tested in IFN-γ ELISA against autologous EBV-LCL overexpressing HLA-DM or HLA-DO and for CMV specificity loaded with CMV antigen. n: number of tested T-cell clones. CMV, cytomegalovirus; EBV, Epstein-Barr virus; IFN, interferon; LCL, lymphoblastic cell lines.

Our vaccination strategy with two epitope variants triggering the same T-cell receptor is very comparable between the different epitope variants, however, it is also quite artificial. Therefore, we sought to analyze whether natural viral infections in humans trigger T-cell responses against both DM-sensitive and DM-resistant epitopes. We generated T-cell libraries^26^ to screen for T-cell responses against EBV and CMV in healthy donors. Briefly, CD4+ T cells were isolated from peripheral blood of eight different healthy human donors, seeded with 250 cells per well and were non-specifically expanded in the presence of irradiated allogeneic PBMCs, PHA and IL-2. After 2–3 weeks of in vitro expansion, all T-cell pools were tested against autologous EBV-LCL overexpressing either HLA-DM or HLA-DO by IFN-γ ELISA. Overexpression of HLA-DM and HLA-DO, respectively, was confirmed by intracellular flow cytometry (online supplemental figure 6). T-cell pools with no or low (OD_(450-570)_<0.5) reactivity were scored as ‘non-reactive’, the remaining pools were assigned to DM-resistant or DM-sensitive based on differential recognition of the HLA-DM or HLA-DO overexpressing EBV-LCL. T-cell pools were considered as DM-sensitive if recognition in the context of HLA-DO was increased by more than 50%. Furthermore, all ‘non-reactive’ T-cell pools were further tested for IFN-γ secretion against HLA-DM and HLA-DO overexpressing EBV-LCL pulsed with CMV antigen. They were again evaluated for their reactivity pattern. The data show that the vast majority of CMV reactive T-cells displayed a DM-resistant phenotype (101 of 103 T-cell pools DM-resistant) (figure 5A), while for EBV 16.5% of the analyzed T-cell pools recognized DM-sensitive antigens (40 of 243 T-cell pools DM-sensitive) (figure 5B).

We further analyzed whether specific T-cell subsets and especially naïve T-cells harbor a higher percentage of T-cells targeting DM-sensitive antigens. Therefore, we isolated naïve (T_N_), effector memory (T_EM_), central memory (T_CM_) and effector memory RA (T_EMRA_) T-cells based on expression of CCR7 and CD45RO and generated T-cell libraries from each subset as described above. Each of the expanded T-cell pools were again tested against HLA-DM or HLA-DO overexpressing autologous EBV-LCL with or without CMV antigen by IFN-γ ELISA. While again T-cell pools against CMV across all subsets were mainly DM-resistant, T-cell pools against EBV recognized DM-sensitive antigen in about 30% of cases (table 3).

**Table 3:** Frequency of DM-sensitive T-cell reactivity among CD4+ T-cell libraries generated from naïve (T_N_), central memory (T_CM_), effector memory (T_EM_) and effector memory RA (T_EMRA_) T-cells

	Epstein-Barr virus	Cytomegalovirus	P value
**T_N_**	31% (56/183)	6% (6/100)	<0.001
**T_CM_**	36% (75/210)	6% (9/162)	<0.001
**T_EM_**	27% (74/274)	0% (0/102)	<0.001
**T_EMRA_**	26% (66/251)	6% (7/122)	<0.001

Depicted are percentages and absolute numbers of T-cell lines with DM-sensitive properties in relation to all reactive T-cell lines. Significance was calculated using Pearson’s χ^2^ test or Fisher’s exact test for small numbers (T_EM_).

In addition we generated CD4+ T-cell clones specific for CMV (n=5) and EBV (n=81) from healthy human donors and tested their reactivity pattern against autologous EBV-LCL overexpressing HLA-DM or HLA-DO with or without loaded CMV antigen. Based on the amount of secreted IFN-γ T-cell clones were assigned to recognize DM-sensitive or DM-resistant antigens. Again, all CMV specific T-cell clones were reactive against DM-resistant antigens, while 40% of the EBV reactive T-cell clones showed DM-sensitive characteristics (figure 5C, D).

## Discussion

CD4+ T-cells play a pivotal role in adaptive immune responses both against tumors and infectious threads. Therefore, processing and presentation of MHC class II restricted antigens and the immunogenicity of the respective epitopes are key parameters for the quality of an immune response. Due to their unique processing behavior relying on the tissue-specific expression of HLA-DO, DM-sensitive antigens might be an interesting option for immunotherapy after aSCT; however, it is unclear whether DM-sensitive antigens exhibit sufficient immunogenicity in vivo. We here show that we can switch epitopes from DM-sensitive to DM-resistant and vice versa by mutating single amino acids within the T-cell epitope, and that both, DM-sensitive and DM-resistant epitope variants were equally capable of activating the same T-cell receptors in vivo. Finally, we could demonstrate that the natural human T-cell repertoire against EBV and CMV targets both DM-sensitive and DM-resistant antigens, although to a different extent.

It has been described for I-A^b^ binding peptides that the four MHC pockets at P1, P4, P6, and P9, that usually bind peptide side chains, are largely empty due to alanine residues at these positions.^24^ Stability of the peptide-MHC complex is preserved due to unique alternate interactions between the peptide and the MHC molecule, especially between the N-terminus of the peptide and the I-A^b^ α-chain as well as between an asparagine at position P7 and the I-A^b^ β-chain. DM-sensitivity has been linked to lower kinetic stability of the MHC-peptide complex.^27^ The wildtype OT-2 epitope of OVA has already reported to be DM-sensitive^22 23^ and this phenotype could be confirmed in our experimental setting. Therefore, we aimed to strengthen the peptide-MHC interaction by mutating one to three amino acids of the epitope without affecting recognition by the T-cell receptor. While mutation of the P6 position strongly affected the predicted affinity from 339 nM to 89 nM, it did not change DM-sensitivity. However, as described by Liu *et al*^24^ tightening the N-terminal end of the peptide by the substitution of alanine to tyrosine not only increased the predicted affinity, but also led to a switch from DM-sensitivity to DM-resistance. In contrast, the DBY epitope already displayed a DM-resistant phenotype and therefore we tried to lower the predicted binding affinity. Here, mainly mutation of the P1 residue was capable of affecting DM-sensitivity without disrupting T-cell recognition. This is in line with the observation that efficient binding of HLA-DM followed by selected peptide exchange requires release of both the P1 anchor and the N-terminal peptide residues as described for human HLA-DM/HLA-DR interaction.^28^

Although we used long peptides instead of full-length proteins, we observed the same behavior of the OT-2 epitope towards H2-M as has been reported for full-length proteins.^22 23^ Nevertheless, it has to be considered that long peptides might be differently processed as compared with full-length proteins and even more so, exogenously added peptides likely follow different routes of processing as compared with endogenously expressed proteins. We so far could not observe any dependency of DM-sensitivity or DM-resistance on the route of antigen application, that is, exogenous or endogenous,^6^ however, further studies demonstrating anti-tumor immunity directed against endogenously expressed DM-sensitive antigens will be needed.

On vaccination of mice with the different epitope variants, we did not observe any significant differences. There is a clear trend towards higher T-cell numbers after vaccination with the DM-resistant OT-2 peptide variants, which might be due to stronger proliferative capacity as suggested by slightly higher Ki-67 staining. After vaccination with DBY epitopes, we observed an earlier increase in T-cell numbers and it might therefore be possible that Ki-67 staining on day 7 was too late to observe discrete differences. Nevertheless, these subtle variations are neither significant nor likely to be of biological relevance. We therefore, propose that DM-sensitive antigens can be as immunogenic as DM-resistant ones. This is also supported by our data showing that re-stimulation of the in vivo expanded T-cells with both peptide variants did not reveal any differences in functional T-cell avidity.

One drawback of the in vivo vaccination part of our study is the usage of T-cell receptor transgenic T-cells. These T-cells express one single antigen specific T-cell receptor with a fixed affinity. This was chosen to be able to directly compare antigenicity of the different peptide variants without influence of a diverse T-cell receptor repertoire. However, we, therefore, cannot account for the initial priming capacity of our peptide variants on the T-cell repertoire. It has been shown that inherent tuning events involving selective expansion of T-cells maintain antigen reactivity in vivo. This suggested that increasing antigenic strength in vivo correlated with reduction in productive T-cell activation.^29^ The authors show that immunization with a low affinity peptide results in high affinity T-cells, whereas on immunization with a high affinity peptide these potent T-cells rapidly progress to the contraction phase of the immune response and are eliminated by apoptosis.^29^ In another study with mutated peptides with a range of MHC class II binding half-lives, it was also shown that short half-lives focuses the T-cell response towards high-affinity clonotypes.^30^ Similar to our data these authors also show that vaccination of mice treated with adoptive transfer of TCR-transgenic T-cells led to comparable numbers of T-cells independent of the stability of the MHC-peptide complex. They also observed that antigen-specific CD4 T-cells accumulated earlier in the lymph nodes of mice vaccinated with high-stability peptides, but that did not influence the overall magnitude of the immune response.^30^ This is also in line with our data showing a trend to higher proliferative capacity on vaccination with DM-resistant variants.

Importantly, immunogenicity of a specific antigen also depends on its capacity to induce initial priming of naïve T-cells. To include this priming phase we analyzed natural immune responses against CMV and EBV in humans. It has already been shown for HLA-B27 restricted epitopes in EBV that immunodominance did not correlate with epitope abundance on antigen presenting cells.^31^ We here found that up to 40% of randomly isolated EBV-specific CD4+ T cell lines display a DM-sensitive phenotype, underlining the immunogenic relevance of DM-sensitive antigens in vivo. Of note, the percentage of T-cells directed against DM-sensitive antigens for EBV varied considerably between 16% and 40%. This might be due to a methodological aspect as single lines of the T-cell libraries do not necessarily consist of only one EBV-reactive T-cell clone. Especially for donors with a high frequency of EBV-reactive T-cells, it might therefore be possible that one T-cell line consists of several different clones leading to loss of differences between HLA-DM and HLA-DO transduced EBV-LCL and resulting in underestimation of the frequency of DM-sensitive antigens. This is also underlined by the higher percentage of T-cells directed against DM-sensitive antigens among the clonally expanded T-cells.

Interestingly, for CMV we rarely found any T-cells with DM-sensitive properties. This might be due to the nature of the primary antigen expressing cell. While for EBV the infected cells are mainly B-cells, which are naturally HLA-DO positive and capable to constantly assemble HLA-peptide complexes from the intracellular antigens, CMV permissive cells are mainly of epithelial origin. As epithelial cells are probably not the main T-cell priming cells, antigens are likely released from dying cells and taken up by surrounding APCs. It is feasible that under these circumstances the immune response is more biased towards DM-resistant epitopes that are capable of forming long-lasting stable HLA-peptide complexes. To use DM-sensitive antigens as targets in anti-tumor immunity, it might therefore be of importance that the malignant cells are capable to present the antigen themselves, that is, tumor cells should be HLA II and HLA-DO/H2-O positive as applicable for most hematological malignancies.^9^

In conclusion, our data provide evidence that DM-sensitive antigens are capable of inducing in vivo immune responses comparable to DM-resistant antigens and underscore their potential for immunotherapy after aSCT.

## Data Availability

All data relevant to the study are included in the article or uploaded as supplementary information.

## References

[1] Schietinger A, Philip M, Liu RB, et al. Bystander killing of cancer requires the cooperation of CD4(+) and CD8(+) T cells during the effector phase. J Exp Med 2010;207:2469–77. 10.1084/jem.20092450 20921286 PMC2964573

[2] Meyer RG, Britten CM, Wehler D, et al. Prophylactic transfer of CD8-depleted donor lymphocytes after T-cell–depleted reduced-intensity transplantation. Blood 2007;109:374–82. 10.1182/blood-2006-03-005769 16940425

[3] Soiffer RJ, Alyea EP, Hochberg E, et al. Randomized trial of CD8+ T-cell depletion in the prevention of graft-versus-host disease associated with donor lymphocyte infusion. Biol Blood Marrow Transplant 2002;8:625–32. 10.1053/bbmt.2002.v8.abbmt080625 12463482

[4] van Balen P, van Bergen CAM, van Luxemburg-Heijs SAP, et al. Cd4 donor lymphocyte infusion can cause conversion of chimerism without GVHD by inducing immune responses targeting minor histocompatibility antigens in HLA class II. Front Immunol 2018;9:3016. 10.3389/fimmu.2018.03016 30619360 PMC6305328

[5] Stevanović S, van Bergen CAM, van Luxemburg-Heijs SAP, et al. Hla class II upregulation during viral infection leads to HLA-DP–directed graft-versus-host disease after CD4+ donor lymphocyte infusion. Blood 2013;122:1963–73. 10.1182/blood-2012-12-470872 23777765

[6] Kremer AN, van der Meijden ED, Honders MW, et al. Endogenous HLA class II epitopes that are immunogenic in vivo show distinct behavior toward HLA-DM and its natural inhibitor HLA-DO. Blood 2012;120:3246–55. 10.1182/blood-2011-12-399311 22889757

[7] Denzin LK, Sant'Angelo DB, Hammond C, et al. Negative regulation by HLA-DO of MHC class II-restricted antigen processing. Science 1997;278:106–9. 10.1126/science.278.5335.106 9311912

[8] Walter W, Scheuer C, Lingnau K, et al. H2-M, a facilitator of MHC class II peptide loading, and its negative modulator H2-O are differentially expressed in response to proinflammatory cytokines. Immunogenetics 2000;51:794–804. 10.1007/s002510000210 10970094

[9] Kremer AN, van der Meijden ED, Honders MW, et al. Human leukocyte antigen-DO regulates surface presentation of human leukocyte antigen class II–Restricted antigens on B cell malignancies. Biol Blood Marrow Transplant 2014;20:742–7. 10.1016/j.bbmt.2014.02.005 24530695

[10] Stumpf AN, van der Meijden ED, van Bergen CAM, et al. Identification of 4 new HLA-DR–restricted minor histocompatibility antigens as hematopoietic targets in antitumor immunity. Blood 2009;114:3684–92. 10.1182/blood-2009-03-208017 19706888

[11] Lazarski CA, Chaves FA, Jenks SA, et al. The kinetic stability of MHC class II:peptide complexes is a key parameter that dictates immunodominance. Immunity 2005;23:29–40. 10.1016/j.immuni.2005.05.009 16039577

[12] Sant AJ, Chaves FA, Jenks SA, et al. The relationship between immunodominance, DM editing, and the kinetic stability of MHC class II:peptide complexes. Immunol Rev 2005;207:261–78. 10.1111/j.0105-2896.2005.00307.x 16181342

[13] Gerbitz A, Sukumar M, Helm F, et al. Stromal interferon-γ signaling and cross-presentation are required to eliminate Antigen-Loss variants of B cell lymphomas in mice. PLoS One 2012;7:e34552. 10.1371/journal.pone.0034552 22479645 PMC3316708

[14] Szyska M, Herda S, Althoff S, et al. A Transgenic Dual-Luciferase Reporter Mouse for Longitudinal and Functional Monitoring of T Cells In Vivo. Cancer Immunol Res 2018;6:110–20. 10.1158/2326-6066.CIR-17-0256 29259004

[15] Griffioen M, van der Meijden ED, Slager EH, et al. Identification of phosphatidylinositol 4-kinase type II as HLA class II-restricted target in graft versus leukemia reactivity. Proc Natl Acad Sci U S A 2008;105:3837–42. 10.1073/pnas.0712250105 18316730 PMC2268788

[16] Fehse B, Kustikova OS, Li Z, et al. A novel 'sort-suicide' fusion gene vector for T cell manipulation. Gene Ther 2002;9:1633–8. 10.1038/sj.gt.3301828 12424616

[17] Griffioen M, van Egmond HME, Barnby-Porritt H, et al. Genetic engineering of virus-specific T cells with T-cell receptors recognizing minor histocompatibility antigens for clinical application. Haematologica 2008;93:1535–43. 10.3324/haematol.13067 18768532

[18] Pfaffl MW. A new mathematical model for relative quantification in real-time RT-PCR. Nucleic Acids Res 2001;29:45e–45. 10.1093/nar/29.9.e45 PMC55695 11328886

[19] Lantz O, Grandjean I, Matzinger P, et al. γ chain required for naïve CD4+ T cell survival but not for antigen proliferation. Nat Immunol 2000;1:54–8. 10.1038/76917 10881175

[20] Gary R, Aigner M, Moi S, et al. Clinical-Grade generation of peptide-stimulated CMV/EBV-specific T cells from G-CSF mobilized stem cell grafts. J Transl Med 2018;16:124. 10.1186/s12967-018-1498-3 29743075 PMC5941463

[21] Robertson JM, Jensen PE, Evavold BD. DO11.10 and OT-II T cells recognize a C-terminal ovalbumin 323-339 epitope. J Immunol 2000;164:4706–12. 10.4049/jimmunol.164.9.4706 10779776

[22] Alfonso C, Williams GS, Han J-O, et al. Analysis of H2-O influence on antigen presentation by B cells. J Immunol 2003;171:2331–7. 10.4049/jimmunol.171.5.2331 12928379

[23] Draghi NA, Denzin LK. H2-O, a MHC class II-like protein, sets a threshold for B-cell entry into germinal centers. Proc Natl Acad Sci U S A 2010;107:16607–12. 10.1073/pnas.1004664107 20807742 PMC2944729

[24] Liu X, Dai S, Crawford F, et al. Alternate interactions define the binding of peptides to the MHC molecule iab. Proc Natl Acad Sci U S A 2002;99:8820–5. 10.1073/pnas.132272099 12084926 PMC124382

[25] Scott D, Addey C, Ellis P, et al. Dendritic cells permit identification of genes encoding MHC class II-restricted epitopes of transplantation antigens. Immunity 2000;12:711–20. 10.1016/S1074-7613(00)80221-6 10894170

[26] Geiger R, Duhen T, Lanzavecchia A, et al. Human naive and memory CD4+ T cell repertoires specific for naturally processed antigens analyzed using libraries of amplified T cells. J Exp Med 2009;206:1525–34. 10.1084/jem.20090504 19564353 PMC2715094

[27] Lazarski CA, Chaves FA, Sant AJ. The impact of DM on MHC class II–restricted antigen presentation can be altered by manipulation of MHC–peptide kinetic stability. J Exp Med 2006;203:1319–28. 10.1084/jem.20060058 16682499 PMC2121212

[28] Anders A-K, Call MJ, Schulze M-SED, et al. Hla-Dm captures partially empty HLA-DR molecules for catalyzed removal of peptide. Nat Immunol 2011;12:54–61. 10.1038/ni.1967 21131964 PMC3018327

[29] Anderton SM, Radu CG, Lowrey PA, et al. Negative selection during the peripheral immune response to antigen. J Exp Med 2001;193:1–12. 10.1084/jem.193.1.1 11136816 PMC2195878

[30] Baumgartner CK, Ferrante A, Nagaoka M, et al. Peptide-Mhc class II complex stability governs CD4 T cell clonal selection. J.i. 2010;184:573–81. 10.4049/jimmunol.0902107 PMC2975033 20007533

[31] Crotzer VL, Christian RE, Brooks JM, et al. Immunodominance among EBV-derived epitopes restricted by HLA-B27 does not correlate with epitope abundance in EBV-transformed B-lymphoblastoid cell lines. J Immunol 2000;164:6120–9. 10.4049/jimmunol.164.12.6120 10843661

