# Supplementary material for: Influence of DM-sensitivity on immunogenicity of MHC class II restricted antigens
Source: J Immunother Cancer. 2021 Jul 15;9(7):e002401. doi: 10.1136/jitc-2021-002401 (PMC8286791; doi:10.1136/jitc-2021-002401)
Supplement: Supplementary data [file jitc-2021-002401supp001.pdf]

Supplemental Table 1: Oligonucleotides used for cloning

H2-M α-chain	H2-Ma_fw_Mlu	NM_010386.4
	CGC *ACGCGT* ACCATGGAGCATGAGCAGAAGTC	
	H2-Ma_rv_Xho	
	CGG *CTCGAG* AAGTCAGTCACCTGAGCACGGTCT	
H2-M β-chain	H2-Mb1_fw_Mlu	NM_010388
	CGC *ACGCGT* ACCATGGCTGCACTCTGGCTGCT	
	H2-Mb1_rv_Xho	
	CGG *CTCGAG* TTACTAATGCCGTCCTTCTGGGTA	
H2-O α-chain	H2-Oa_fw_Mlu	NM_008206.2
	CGC *ACGCGT* GCAATGGTCCTTTTTGTGGAGCT	
	H2-Oa_rv_Xho	
	CGG *CTCGAG* AAGTTACCTGCGGATACTGGGCC	
H2-O β-chain	H2-Ob_fw_Mlu	NM_010389
	CGC *ACGCGT* AGAATGGGCGCTGGGAGGGCCCC	
	H2-Ob_rv_Xho	
	CGG *CTCGAG* TTACTAGGGTTGAGAATGGAGACT	
I-A^{b} α-chain	BamH1_I-Ab alpha_fw	NM_010378
	CGC *GGATCC* AGGATGCCGCGCA GCAGAGCTC	
	EcoR1_I-Ab alpha_rv	
	CCG *GAATTC* GACTCATAAAGGCCCTGGGTGTC	
I-A^{b} β-chain	Nco1_I-Ab beta_fw	NM_207105

	TATCAC *CCATGG* CTCTGCAGATCCCCAG	
	Not1_I-Ab beta_rv	
	GAAT *GCGGCCGC* GAGTCACTGCAGGAGCCCTGC	
HLA-DM α-chain	Mlu1_HLA-DMA_fw	NM_006120.4
	CGC *ACGCGT* GGT ATGGGTCATGAACAGAACCAAG	
	Xho1_HLA-DMA _rv	
	CCG *CTCGAG* ACC TCAGTCACCTGAGCAAGGCTT C	
HLA-DM β-chain	Mlu1_HLA-DMB_fw	NM_002118.5
	CGC *ACGCGT* AGCATGATCACATTCCTGCCGCTG	
	Xho1_HLA-DMB _rv	
	CCG *CTCGAG* CCTCTAGGAAATGTGCCATCCTTC	
HLA-DO α-chain	Mlu1_HLA-DOA_fw	NM_002119.4
	CGC *ACGCGT* GTAATGGCCCTCAGAGCAGGGC	
	Xho1_HLA-DOA _rv	
	CCG *CTCGAG* TCATTACCTGGGGACACTGGAC	
HLA-DO β-chain	Mlu1_HLA-DOB_fw	NM_002120.4
	CGC *ACGCGT* AGAATGGGTTCTGGGTGGGTCC	
	Xho1_HLA-DOB _rv	
	CCG *CTCGAG* ACCTTAGCATGACTGAGGGAGC	

Oligonucleotides are depicted from 5’ to 3’ end. Restriction enzyme sites are in italics and start or stop codons are underlined.

Supplemental Table 2: Oligonucleotides for quantitative RT-PCR

18S rRNA	mu18S_fw
	CGCCGCTAGAGGTGAAAT
	mu18S_rv
	CGAACCTCCGACTTTCGT
H2-M α-chain	H2-Ma_5_fw
	AGCATGAGCAGAAGTCAGGAG
	H2-Ma_145_rv
	ATAGAGTGTGCCGGAATGTG
H2-M β-chain	H2-Mb_566_fw
	TCTACACCTGCGTGGTTCAG
	H2-Mb_658_rv
	AGACCTTCACTGTCTGGATGG
H2-O α-chain	H2-Oa_73_fw
	TCCTTTTTGTGGAGCTGGTC
	H2-Oa_199_rv
	GCGTCGTAAGATTGGTAGAAGG
H2-O β-chain	H2-Ob_114_fw
	TCATGAGGCTGGATTCCTTC
	H2-Ob_195_rv
	AAGTAACAGTCCGCCTTTGC
