# Supplementary material for: Influence of DM-sensitivity on immunogenicity of MHC class II restricted antigens
Source: J Immunother Cancer. 2021 Jul 15;9(7):e002401. doi: 10.1136/jitc-2021-002401 (PMC8286791; doi:10.1136/jitc-2021-002401)
Supplement: Supplementary data [file jitc-2021-002401supp002.pdf]

Supplement 1: H2-M/-O and I-A^b Expression in 291PC und A20

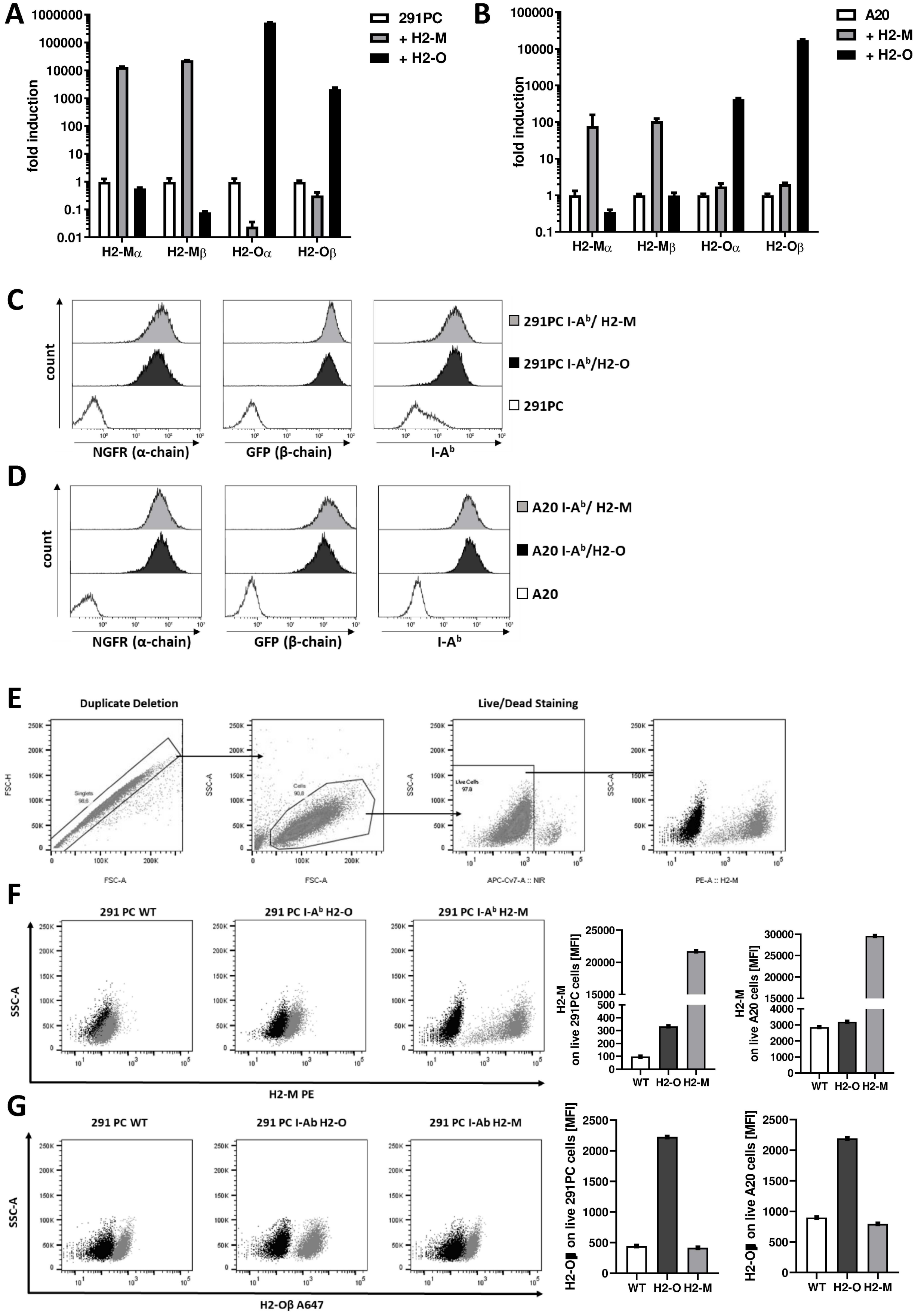

Supplemental Figure 1: Expression of H2-O and H2-M in 291PC and A20 cell lines

Overexpression of H2-M and H2-O after retroviral transduction in 291PC (A) and A20 (B) cell lines was verified by qPCR. Gene expression was normalized to expression of 18S ribosomal RNA and is depicted as fold induction as compared to non-transduced cell lines. Overexpression of H2-M and H2-O at the protein level was verified by measurement of the co-translational marker gene (ΔNGFR for the α-chain and GFP for the β-chain) by flow cytometry for 291PC (C) and A20 (D) cells. Expression of the I-A^b restriction molecule was measured on the cell surface by flow cytometry for 291PC (C) and A20 (D) cells. Intracellular protein expression of H2-M (F) and H2-O (G) in 291PC or A20 cells overexpressing either H2-M or H2-O was verified by direct measurement in flow cytometry. The gating strategy using duplicate deletion and dead cell deletion by NIR staining is depicted in E. Shown are representative dot plots as well as a data summary displaying mean fluorescence intensity (MFI) of the respective antibody. Black: without H2-M/-O antibody [FMO]; grey: with H2-M/-O antibody; white: wildtype control.

Supplement 2: Switching DM-phenotype for OT-2 and DBY using 291PC cells

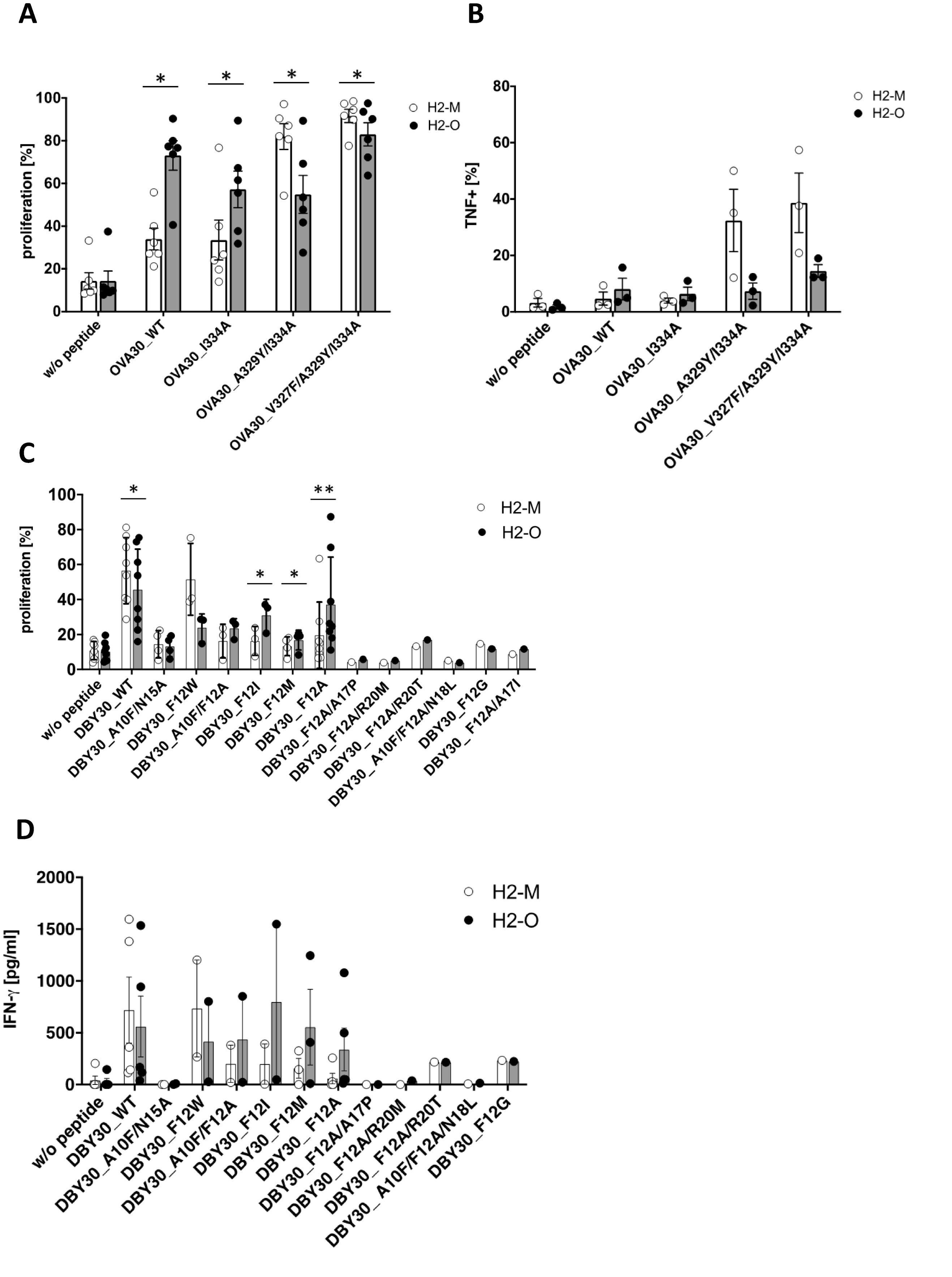

Supplemental figure 2: Switching DM-phenotype for OT-2 and DBY using 291PC cells

CD4+TCRvα2/β5+ T-cells derived from OT-2 mice were stained with CFSE and cocultured with irradiated 291PC cells transduced with H2-M or H2-O and loaded with the indicated peptides. Proliferation was measured on day 5 using flow cytometry (A). (B) Intracellular TNF production was measured after coculture with 291PC cells on day 3.

CD4+TCRvβ6+ T-cells derived from Marilyn mice were stained with CFSE and cocultured with irradiated 291PC cells transduced with H2-M or H2-O and loaded with the indicated peptides. Proliferation was measured on day 3 using flow cytometry (C). (D) IFN-γ secretion was measured by ELISA after coculture with 291PC cells by day 2.

Significance was calculated using paired t-test. *: $p<0.05$, **: $p<0.01$

Supplemental material BMJ Publishing Group Limited (BMJ) disclaims all liability and responsibility arising from any reliance placed on this supplemental material which has been supplied by the author(s)

Supplement 3: T-cell kinetics after vaccination with OT-2 or DBY epitope.

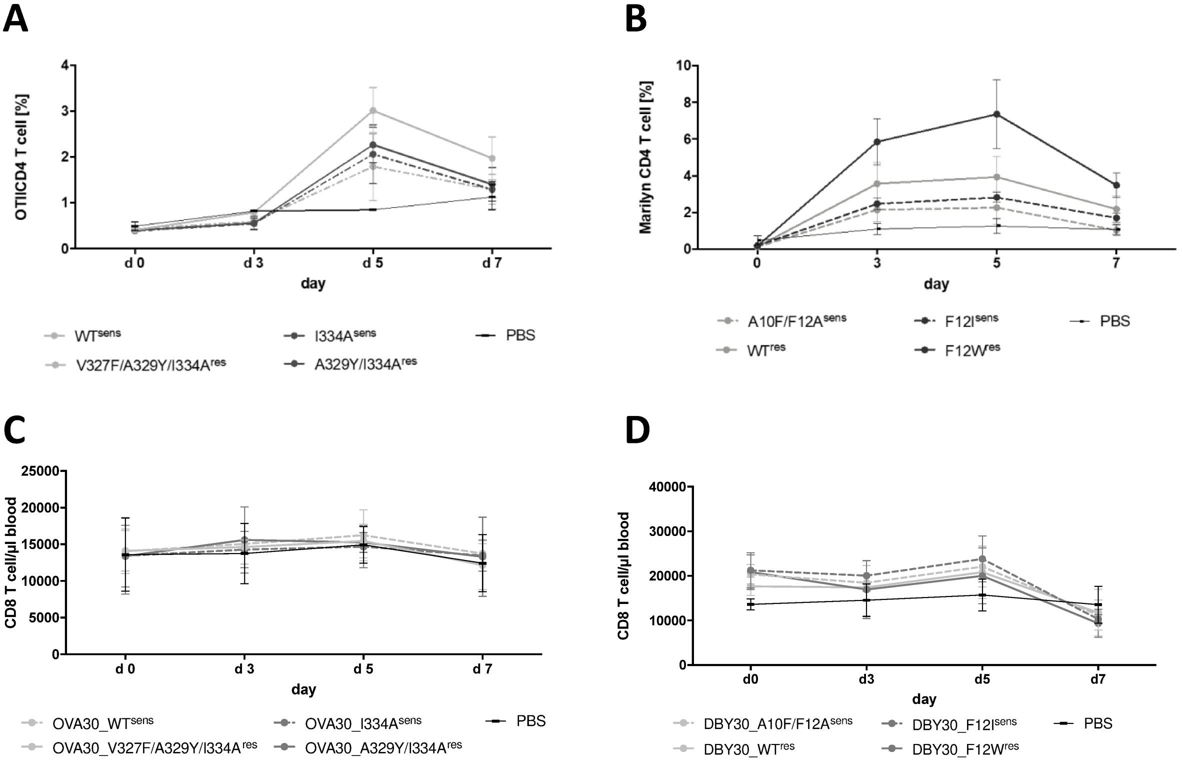

Supplemental figure 3: T-cell kinetics after vaccination with OT-2 or DBY epitope.

CD4+TCRvα2/β5+ OT-2 T-cells were injected i.p. in C57BL/6 mice and mice were vaccinated with the indicated peptides the next day. Frequencies of the CD4+TCRvα2/β5+ T-cells, total CD4 and CD8 T-cells were measured in peripheral blood of treated mice. Depicted are percentages of specific (TCRvα2/β5+) among total CD4 T-cells (A) and total CD8 T-cells (C). Data show means ± SEM (n=3-4/group).

C57BL/6 mice were injected i.p. with in vitro expanded CD4+TCRvβ6+ Marilyn-derived T-cells and subsequently vaccinated with the indicated peptides. Frequencies of the CD4+TCRvβ6+/CD45.1+, total CD4 and CD8 T-cells were measured in peripheral blood of treated mice (n=3-4/group). Depicted are percentages of specific (TCRvα2/β5+) among total CD4 T-cells (B) and total CD8 T-cells (D). Data show means ± SEM (n=3-4/group).

Supplement 4: *In vivo* expansion of T-cells after vaccination with DM-sensitive or -resistant peptides using CpG as adjuvant

A

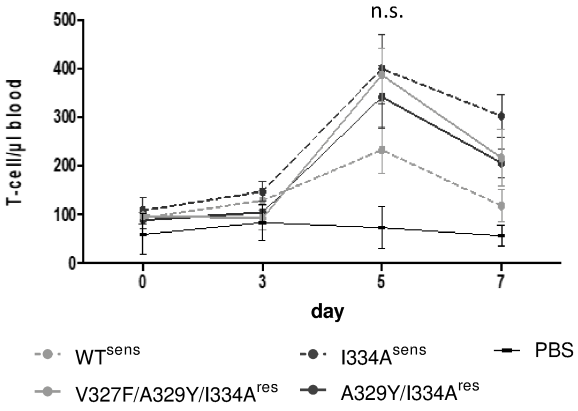

B

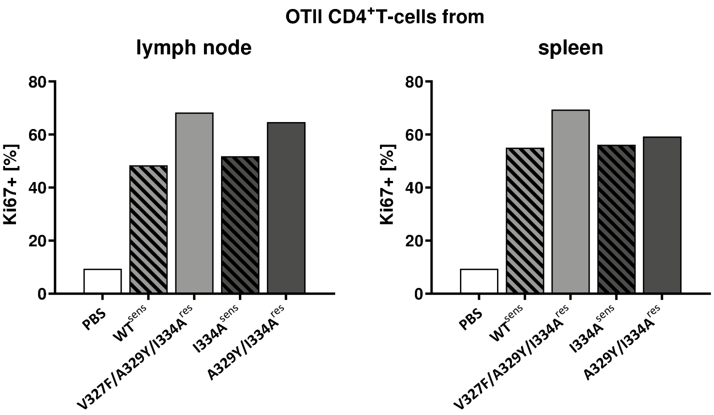

Supplemental Figure 4: *In vivo* expansion of T-cells after vaccination with DM-sensitive or -resistant peptides using CpG as adjuvant

(A) CD4⁺TCRvα2/β5⁺ T-cells were injected i.p. in C57BL/6 mice and mice were subsequently vaccinated with the indicated peptides using CpG as adjuvant. Frequencies of the CD4⁺TCRvα2/β5⁺ T-cells were measured in peripheral blood of the mice. (B) Splenocytes and lymph nodes were isolated from mice after vaccination and i.p. transfer of OT-2 T-cells using CpG as adjuvant. Proliferative capacity of CD4⁺TCRvα2/β5⁺ T-cells was measured by intracellular Ki-67 staining. n=3-4mice/group

Supplemental Figure 5: *Ex vivo* avidity of *in vivo* primed T-cells against DM-sensitive or DM-resistant peptides

A

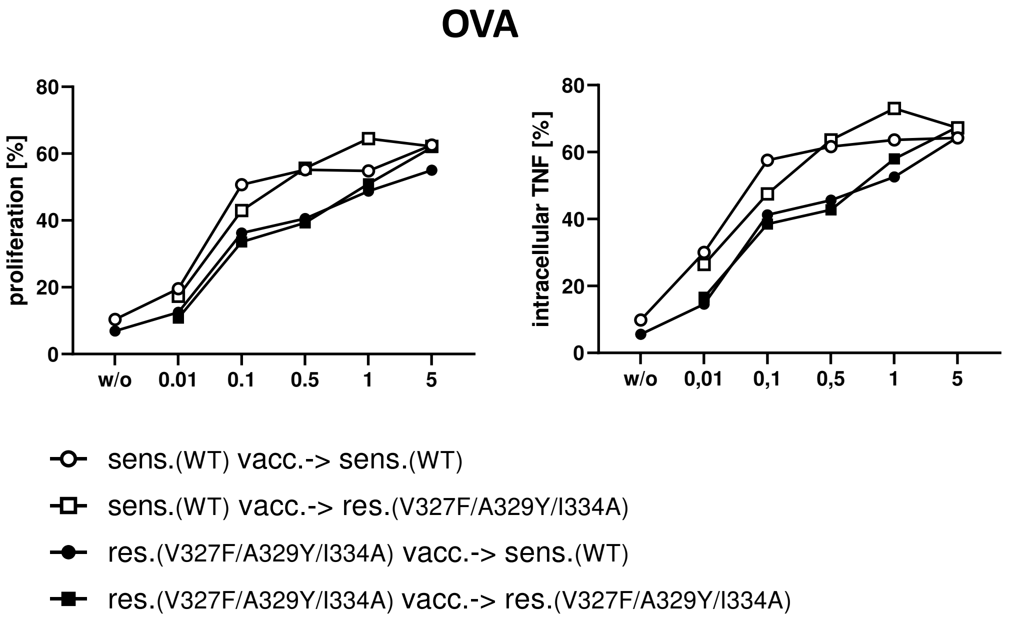

B

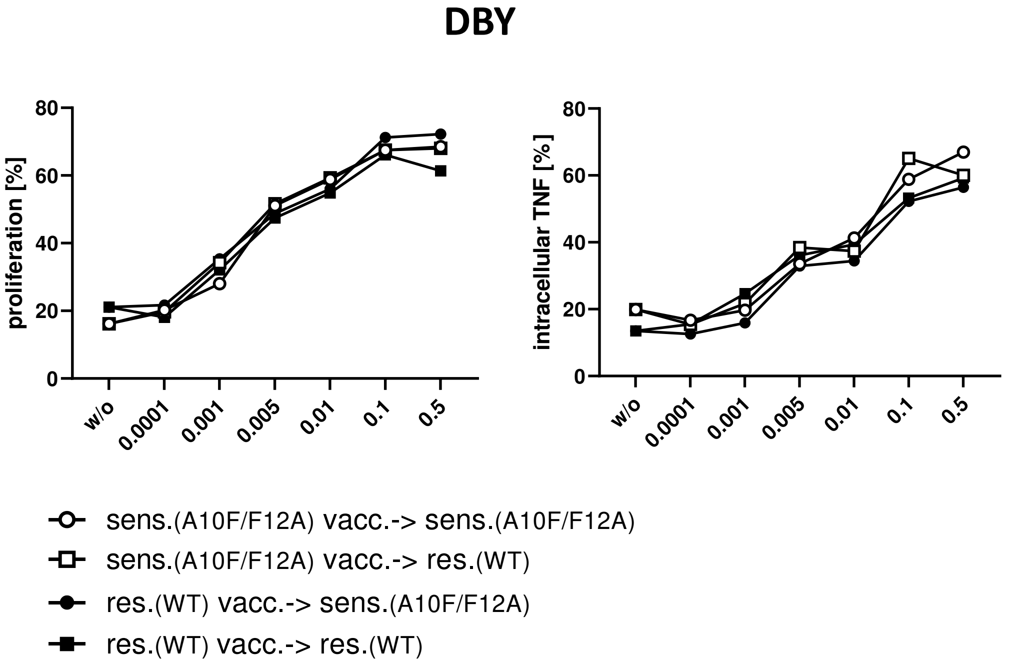

Supplemental Figure 5: ***Ex vivo*** **avidity of** ***in vivo*** **primed T-cells against DM-sensitive or DM-resistant peptides**
OVA (A) or DBY (B) specific T-cells were isolated from spleens of vaccinated mice on day 7 after T-cell transfer and retested against the original vaccination peptide as well as the counterpart peptide. For each combination proliferation and intracellular TNF levels upon stimulation with titrated concentrations of the individual peptides was tested.

Supplemental Figure 6: Expression of HLA-DM and –DO in transduced EBV-LCL

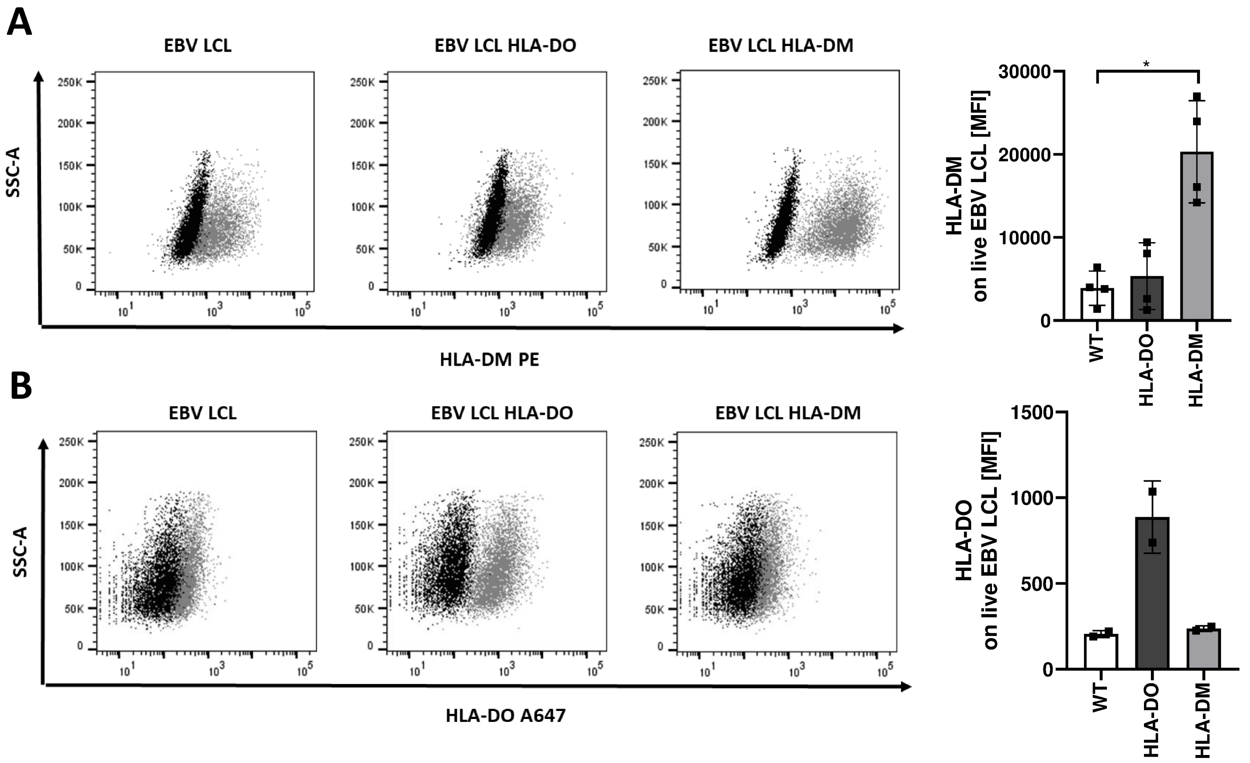

Supplemental Figure 6: Expression of HLA-DM and –DO in transduced EBV-LCL
Expression of HLA-DM (A) and HLA-DO (B) in EBV-LCL overexpressing either HLA-DM or –DO was measured by intracellular flow cytometric staining. Shown are representative dot plots as well as a data summary displaying mean fluorescence intensity (MFI) of the respective antibody. Black: without HLA-DM/-DO antibody [FMO]; grey: with HLA-DM/-DO antibody; white: wildtype control; n=4
